# Analysis of Contractile Function of Permeabilized Human Hypertrophic Cardiomyopathy Multicellular Heart Tissue

**DOI:** 10.3389/fphys.2019.00239

**Published:** 2019-03-28

**Authors:** Nico Kresin, Sabrina Stücker, Elisabeth Krämer, Frederik Flenner, Giulia Mearini, Julia Münch, Monica Patten, Charles Redwood, Lucie Carrier, Felix W. Friedrich

**Affiliations:** ^1^ Institute of Experimental Pharmacology and Toxicology, Cardiovascular Research Center, University Medical Center Hamburg-Eppendorf, Hamburg, Germany; ^2^ DZHK (German Centre for Cardiovascular Research), Partner Site Hamburg/Kiel/Lübeck, Hamburg, Germany; ^3^ University Heart Center Hamburg, Hamburg, Germany; ^4^ Radcliffe Department of Medicine, University of Oxford, John Radcliffe Hospital, Oxford, United Kingdom

**Keywords:** myofilament, Ca^2+^ sensitivity, hypertrophic cardiomyopathy, *MYBPC3*, epigallocatechin-3-gallate, NanoString nCounter

## Abstract

**Background**: Many forms of hypertrophic cardiomyopathy (HCM) show an increased myofilament Ca^2+^ sensitivity. This observation has been mainly made in HCM mouse models, myofilament systems, and cardiomyocytes. Studies of multicellular tissues from patients with different HCM-associated gene mutations are scarce. We investigated Ca^2+^ sensitivity in multicellular cardiac muscle strips of HCM patients. We furthermore evaluated the use of epigallocatechin-3-gallate (EGCg), a Ca^2+^ desensitizer.

**Methods**: After strip isolation from cardiac tissues with single (*MYBPC3*, *MYH7)* or double heterozygous mutations (*MYBPC3/FLNC, MYH7/LAMP2, MYBPC3/MYH7*) and permeabilization, we performed contractility measurements ±EGCg. We furthermore evaluated gene expression with a customized heart failure gene panel using the NanoString technology.

**Results**: F_max_ tended to be higher in HCM than in non-failing (NF) control strips and in single than in double heterozygous strips. Ca^2+^ sensitivity was higher by trend in most HCM vs. NF strips and by trend in tissues with double vs. single heterozygous mutations. EGCg desensitized myofilaments to Ca^2+^ in most of the strips and tended to induce a more pronounced shift in strips with truncating than missense or single than double heterozygous mutations. Gene expression analysis revealed lower *ATP2A2*, *PPP1R1A,* and *FHL2* and higher *NPPA*, *NPPB, COL1A1, CTGF,* and *POSTN* marker levels in HCM than in NF tissues*. NPPA*, *NPPB*, *ACTA1*, *CTGF*, *COL1A1*, and *POSTN* levels were higher in tissues with missense than truncating mutations.

**Conclusion**: We report an increased myofilament Ca^2+^ sensitivity in native multicellular cardiac HCM strips, which by trend was more pronounced in samples with double heterozygous mutations. EGCg could have differential effects depending on the underlying genetic status (single vs. double heterozygous) and type (missense vs. truncating).

## Introduction

Hypertrophic cardiomyopathy (HCM) is the most frequent genetic disease of the myocardium and is primarily caused by mutations coding for sarcomeric proteins (Friedrich and Carrier, 2012; Maron et al., 2014; Ho et al., 2015). The most frequently mutated genes are *MYBPC3,* encoding cardiac myosin-binding protein C, and *MYH7,* encoding β-myosin heavy chain (Walsh et al., 2017). HCM is characterized by asymmetric left ventricular hypertrophy, diastolic dysfunction, and myocardial disarray (Elliott et al., 2008). Current pharmacological treatment of HCM is mainly based on beta-adrenoceptor (AR, beta blockers) and Ca^2+^ channel antagonists, which improve clinical symptoms, in part prevent arrhythmias and recover diastolic dysfunction by extending left ventricular (LV) filling time and reducing outflow tract obstruction (Maron et al., 2003; Gersh et al., 2011; Spoladore et al., 2012; Hamada et al., 2014; Tardiff et al., 2015). An attractive concept for many forms of HCM is an increased myofilament Ca^2+^ sensitivity. This could contribute to compromised diastolic relaxation causing remaining actin-myosin interaction at low diastolic Ca^2+^ concentrations and arrhythmias (Morimoto et al., 1998; Baudenbacher et al., 2008). Interventions decreasing this myofilament Ca^2+^ sensitivity may be appealing for the treatment of HCM and improvement in symptoms (Jagatheesan et al., 2007; Alves et al., 2014; Tardiff et al., 2015). These observations have mainly been made in genetically engineered HCM mouse models, reconstituted myofilament systems (Tardiff et al., 1999; Cazorla et al., 2006; Pohlmann et al., 2007; Morimoto, 2008; Vignier et al., 2009; Fraysse et al., 2012; Alves et al., 2014; Barefield et al., 2014; Wijnker et al., 2016), and in isolated cardiac cells of human HCM samples (Jacques et al., 2008; van Dijk et al., 2009, 2012). We previously reported contractile data from cardiac strips of HCM patients with mutations in the most frequently mutated HCM gene *MYBPC3*, but to the best of our knowledge, studies of multicellular tissues from patients with different HCM-associated gene mutations have not been reported so far. We therefore investigated myofilament Ca^2+^ sensitivity in native multicellular cardiac muscle strips derived from septal myectomies of patients with different HCM gene mutations. We furthermore evaluated the potential use of epigallocatechin-3-gallate (EGCg), the major catechin in green tea and a known Ca^2+^ desensitizer.

## Materials and Methods

### Human Samples

We received cardiac tissue of seven HCM patients carrying single heterozygous mutations in *MYBPC3* (3) or *MYH7* (1) and double heterozygous mutations [*MYBPC3/FLNC* (1), *MYH7/LAMP2* (1), *MYH7/MYBPC3* (1)], who underwent septal myectomy due to outflow tract obstruction (Table 2) in a completely anonymized way. Control donor tissues were from non-failing human heart tissues not suitable for transplantation (*n* = 1, control for contractile function measurements) or from donors who did not die from cardiac disease but of another cause (*n* = 7 for gene expression analysis). All samples were immediately frozen and stored in liquid nitrogen. An approval by and with the Standing Ethics Committee is and was not required for our study since all data obtained and used in this study were de-identified. No identifying information regarding our patients or donors’ identities was included in the manuscript nor do we have concerns that the anonymity cannot be maintained in the way the data are presented. We have written informed consent from all our patients and donors regarding the use of acquired tissue or other materials such as but not limited to clinical data or blood samples for scientific research in accordance with state laws and ethics. No material was used or included without that written informed consent from the patients or donors. This study is in accordance with the Code of the Ethics Committee of Hamburg and the Code of Ethics of the World Medical Association (Declaration of Helsinki). In summary, ethics approval was not required as per our institution’s guidelines and national regulations.

### Skinned Ventricular Trabeculae Force Measurements

Cardiac strips of 2.42 ± 0.62 mm in length, 0.45 ± 0.08 mm in width, and 0.18 ± 0.06 mm^2^ in cross-sectional area (CSA), calculated by 2πr^2^ assuming a circular shape, were isolated from human cardiac tissues (*n* = 4–25/group) and frozen in liquid nitrogen until further analysis. Before contractile analysis, strips were permeabilized in a pCa 9 EGTA-buffer (Kooij et al., 2010; Stoehr et al., 2014; Friedrich et al., 2016) containing 1% Triton X-100 at 4°C for 18 h. The following day strips were either used directly for measurements or stored at −20°C in a 50% glycerol/relaxing solution containing protease inhibitors (EDTA-free, complete tablets, mini, Roche). A fiber test system (1400A; Aurora Scientific) was used to evaluate the contractile function of cardiac strips. Sarcomere length could not be determined reliably in skinned HCM strips due to myocardial and myofilament disarray. Therefore, after mounting strips between a force transducer and a length controller and stretching until slack length, they were stretched another 10% of length. For maximum force measurements, strips were kept in pCa 9 to achieve full relaxation and were then moved to pCa 4.5 until maximal force development was reached, as reported before (Friedrich et al., 2016; Stucker et al., 2017). For force-Ca^2+^ curves, strips were exposed to increasing Ca^2+^ concentrations from pCa 9 to pCa 4.5 in EGTA-buffer, and force development was measured in each pCa solution. Measurements were repeated in the presence of 30 μM epigallocatechin-3-gallate (EGCg, Sigma Life Sciences) after 5 min preincubation in pCa 9 (Flenner et al., 2016; Friedrich et al., 2016; Stucker et al., 2017). To exclude time-dependent loss of force, EGCg was tested first and a control measurement was performed 5 min after EGCg washout in every second measurement. Each strip was measured pair wisely (paired analysis baseline vs. intervention), and the length of the strip was not changed in between. Thereby, each strip served as its own control. Data were analyzed with the Hill equation (Hill et al., 1980), with pCa_50_ as the free Ca^2+^ concentration which produces 50% of the maximal force and nH representing the Hill coefficient. The pCa_50_ represents the measure of myofilament Ca^2+^ sensitivity.

### RNA Isolation and Expression Analysis With the NanoString nCounter^®^ Elements

Total RNA was extracted from cardiac tissues using the SV Total RNA Isolation kit (Promega). For gene expression analysis, we used a customized NanoString’s nCounter^®^ Elements TagSet panel of 27 genes coding for proteins regulated in hypertrophy/heart failure, including Ca^2+^ and K^+^ handling proteins (Prondzynski et al., 2017; Singh et al., 2017; Braumann et al., 2018). About 50 ng of each sample were hybridized to target-specific capture and reporter probes at 67°C overnight (16 h) according to manufacturer’s instructions. Samples were cooled down at 4°C, filled up with 15 μl H_2_O, and loaded into the NanoString cartridge, and the nCounter Gene Expression Assay was started immediately. Raw data were analyzed with nCounter^®^ Sprint Profiler including background subtraction using negative controls and normalization to five housekeeping genes (*ABCF1, CLTC, GAPDH, PGK1,* and *TUBB*). Data represented the mean of normalized counts and were expressed as fold-change. We selected genes that were lower than 0.8-fold and higher than 1.25-fold dysregulated in cardiomyopathy.

### Statistical Analysis

Data were expressed as mean ± SEM. Comparisons were performed by paired Student’s *t*-test (baseline vs. EGCg) and by mixed-effect model analysis followed by Dunnett multiple comparison post-test when analyzing all groups as indicated in the figure legends. For subgroup analyses (single vs. double heterozygous mutations, missense vs. truncating, *MYH7* vs. *MYBPC3*), a qualitative comparison was performed. Concentration response curves were fitted to the data points, and force-pCa relationship comparison was done by using extra sum-of-squares *F*-test (GraphPad, Prism 8). A value of *p* < 0.05 was considered statistically significant.

## Results

### Patients’ Characteristics

HCM patients of the study had undergone septal myectomy due to LV outflow tract obstruction. Echocardiographic and clinical data of the patients are given in Table 1. Patients presented with NYHA states 1.5–3.0 (mean 2.5), preserved ejection fraction (*EF* > 60%), and increased septum wall thickness (mean 28 ± 7 mm). Most of them had a high LV transaortic pressure gradient (mean 87 ± 34 mm Hg), a systolic anterior motion (SAM) of the mitral valve, differing grades of mitral valve insufficiency, and diastolic dysfunction. Some had encountered ventricular tachycardia and syncope and were equipped with an ICD. Medication consisted of ACE inhibitors, beta blockers, and Ca^2+^ channel antagonists.

**Table 1:** Echocardiographic and clinical data of the patients.

Pat. #	NYHA	EF (%)	Septum (mm)	Gradient max	SAM	MI	LA (mm)	DD	VT	ICD	LGE yes/no	Medication	Score	TNT	CK	proBNP	Comment
1	1.5	>60	26	85	1	1	51	2	Yes	Yes	Yes	ACEI	4.0	5	101	6156	
2	2.5	>60	38	65	1	2.5	37	0	Yes	Yes	Yes	Verapamil	8.9	16	89	3449	FA SCD
3	3	>60	21	110	1	2.5	43	2	0	No	n.d.	BB	2.94	10	79	1138	
4	2.5	>60	24	60	1	1	48	2	Yes	Yes	n.d.	BB	5.64	63	143	2543	Syncope
5	2.5	>60	26	110	1	1.5	40	0	0	No	n.d.	BB	4.31	3	48	1517	
6	2.5	>60	24	140	1	1	60	2	0	No	0	BB	6.53	10	57	2622	
7	2.5	>60	39	45	1	2	36	0	Yes	Yes	n.d.	BB	14.3	88	104	5851	Syncope
Mean±SD	**2.5±0.45**	**>60**	**28±7**	**87±34**		**1.6±0.7**	**45±9**	**4/7**	**4/7**	**4/7**			**6.7±3.9**	**28±34**	**89±32**	**3325±1982**	

ACEI, ACE-inhibitor; AF, Atrial fibrillation; BB, Beta blocker; CK, Creatine kinase; DD, Diastolic dysfunction; EF, Ejection fraction; FA SCD, Family Anamnesis Sudden Cardiac Death; ICD, Internal Cardioverter-Defibrillator; LA, Left atrium; LGE, Late gadolinium enhancement; MI, Mitral valve insufficiency; NYHA, New York Heart Association classification of heart failure status; proBNP, pro brain natriuretic peptide SAM, Systolic anterior motion of the mitral valve; TNT, Troponin T; VT, Ventricular tachycardia; Score indicates 5-year risk for sudden cardiac death: >6% ICD implantation indicated.

### Mutation Characteristics

Patients had either single heterozygous mutation in *MYBPC3* (*n* = 3) or *MYH7* (*n* = 1), or double heterozygous mutations [*MYBPC3/FLNC* (*n* = 1), *MYH7/LAMP2* (*n* = 1), *MYBPC3*/*MYH7* (*n* = 1)]. Two mutations are truncating, and the rest are missense mutations. *In silico* analysis using the prediction programs Mutation Taster^1^ and PolyPhen-2^2^ classified most of the mutations as disease causing or possibly damaging (Table 2).

**Table 2:** Mutation characteristics.

Pat. #	Mutation	Genetic Status	Mutation Type	Protein Consequence	Mutation Taster	PolyPhen
1	*MYBPC3* (c.1358dupC)	Single heterozygous	Truncating	p.Val454CysfsX21	Disease causing (1)	__
2	*MYBPC3* (c.1960C>T)	Single heterozygous	Missense	p.Arg654Cys	Disease causing (0.99)	Possibly damaging (0.843)
3	*MYBPC3* (c.2308G>A)	Single heterozygous	Truncating	p.Asp770SerX98	Disease causing (0.99)	Possibly damaging (0.953)
4	*MYH7* (c.598G>C)	Single heterozygous	Missense	p.Ala200Pro	Disease causing (0.99)	Possibly damaging (0.842)
5	*MYBPC3/FLNC* (c.2234A>G; c.3004C>T)	Double heterozygous	Missense	*MYBPC3*: *p.Asp745Gly/pAsp745del25* *FLNC*: *p.Arg1002Trp*	*MYBPC3*: Disease causing (0.99) *FLNC*: Disease causing (0.96)	*MYBPC3*: Possibly damaging (0.826) *FLNC*: Benign: 0.022
6	*MYH7/LAMP2* (c.1988G>A; c.277G>A)	Double heterozygous	Missense	*MYH7*: *p.Arg663His* *LAMP2*: *p.Gly93Arg*	*MYH7*: Disease causing (0.92) *LAMP2*: Disease causing (0.99)	*MYH7*: Possibly damaging (0.628) *LAMP2*: Possibly damaging (0.844)
7	*MYBPC3/MYH7* (c.1293C>T; c.1432A>G)	Double heterozygous	Missense	*MYBPC3*: *p.Asp431Asp* *MYH7*: *p.Ile478Val*	*MYBPC3*: Disease causing (1) *MYH7*: Disease causing (0.99)	*MYBPC3*: No data *MYH7*: Benign (0.002)

*ACTN2*, Actinin Alpha 2; *FLNC*, Filamin C; *LAMP2*, Lysosome-associated membrane protein 2; *MYBPC3*, Myosin-binding protein C, cardiac-type; *MYH7*, Myosin heavy chain 7; for Mutation Taster/Poly Phen scores please refer to: http://www.mutationtaster.org, http://genetics.bwh.harvard.edu/pph2/.

## Varying Maximal Force Development in HCM Strips

**Figure 1:**
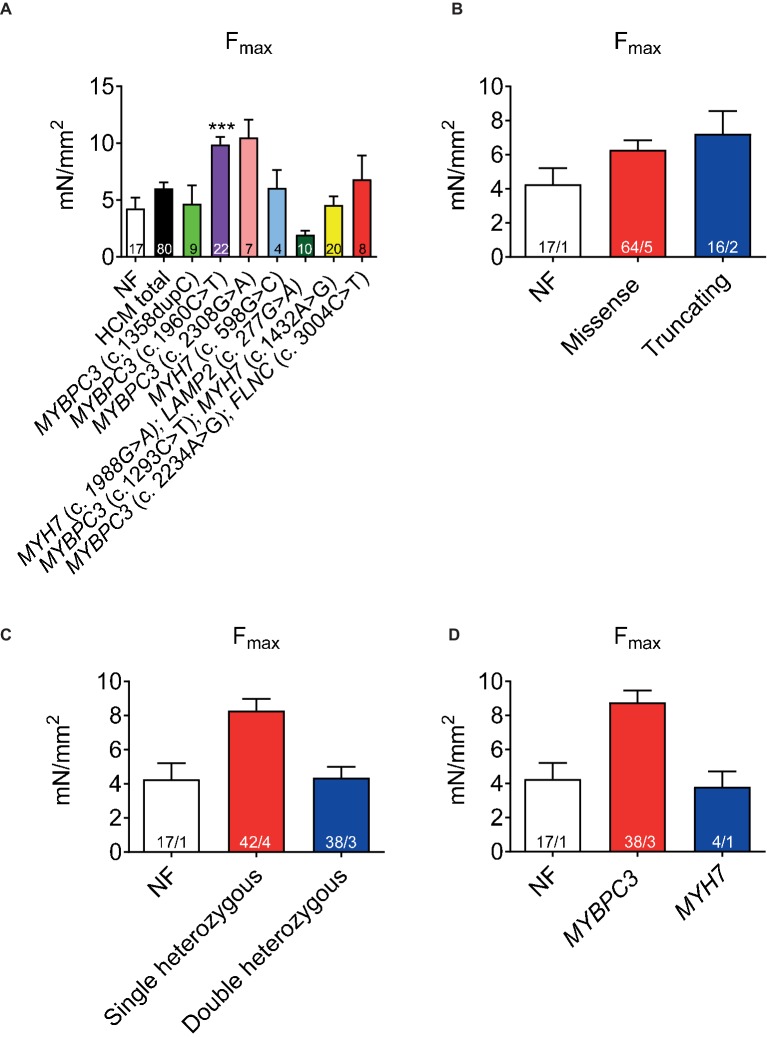
Force development of permeabilized cardiac muscle strips isolated from seven HCM patients with either single heterozygous (*MYBPC3*, *MYH7*) or double heterozygous mutations (*MYH7/LAMP2*, *MYBPC3/MYH7*, *MYBPC3/FLNC*). Maximal force development (F_max_) related to cross-sectional area in non-failing control vs. HCM strips **(A)**, grouped in missense or truncating **(B)**, single or double heterozygous mutations **(C)**, and classified according to a pure *MYBPC3* or *MYH7* genotype **(D)**. Values are related to non-failing control (NF) strips. Data are expressed as mean ± SEM. **p < 0.01 and ***p < 0.001 vs. NF, mixed-effect model analysis followed by Dunnett multiple comparison post-test; for the different subgroup analyses, only qualitative comparisons were performed. *n* = number of strips; *n*/*N* = number of strips/number of patients as depicted in the bars.

Functional implications of the HCM mutations were evaluated by contractile function measurements of permeabilized cardiac muscle strips. Force development (F_max_) related to cross-sectional area showed a trend to higher F_max_ in HCM (black bar, Figure 1A) than in the NF strips. It was higher in one sample carrying a single heterozygous *MYBPC3* mutation (Figure 1A). F_max_ did not differ between tissues with missense or truncating mutations (Figure 1B), whereas it tended to be higher in tissues with single than double heterozygous mutations or NF (Figure 1C) and in tissues with an *MYBPC3* vs. *MYH7* genotype (Figure 1D).

### Myofilament Ca^2+^ Sensitivity Is Higher in HCM Than in NF Strips

**Figure 2:**
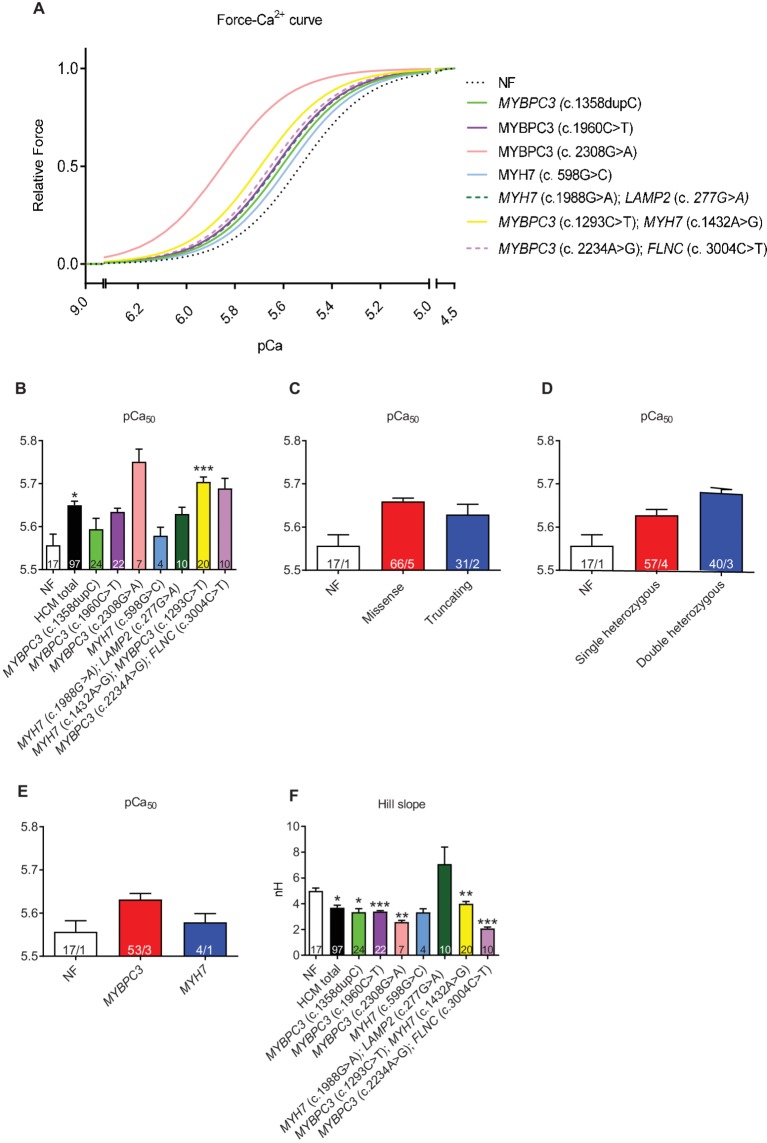
Force-Ca^2+^ relationship of permeabilized cardiac muscle strips isolated from seven HCM patients with either single heterozygous (*MYBPC3*, *MYH7*) or double heterozygous mutations (*MYH7/LAMP2*, *MYBPC3/MYH7*, *MYBPC3/FLNC)*. **(A)** Force-Ca^2+^ relationship curves in human strips, Hill slope set to 3. **(B)** pCa_50_ representing the negative logarithm of the Ca^2+^ concentration needed for half-maximal activation. **(C–E)** pCa_50_ in strips grouped according to mutation type (missense or truncating), to genetic status (single or double heterozygous mutations), and to a pure *MYBPC3* or *MYH7* genotype. **(F)** nHill coefficient (Hill slope). Values are related to non-failing control (NF) strips. Data are expressed as mean ± SEM. *p < 0.05, **p < 0.01, and ***p < 0.001 vs. NF, mixed-effect model analysis followed by Dunnett multiple comparison post-test; for the different subgroup analyses, only qualitative comparisons were performed; concentration response curves were fitted to the data points and curve comparison was done by using extra sum-of-squares F-test. *n* = number of strips; *n*/*N* = number of strips/number of patients as depicted in the bars.

Force-Ca^2+^ curves revealed a higher myofilament Ca^2+^ sensitivity (=higher pCa_50_) in HCM vs. NF strips (pCa_50_ NF: 5.57; pCa_50_ HCM_total_: 5.65; Figures 2A,B). This was independent of the mutation type (Figure 2C), whereas pCa_50_ was by trend higher in muscle strips with double than single heterozygous mutations (Figure 2D) and in *MYBPC3* than *MYH7* genotypes (Figure 2E). Hill slope (N_H_) as the indicator of cooperativity was lower or showed a trend to lower values in almost all HCM in comparison to NF strips except for strips carrying the double *MYH7*/*LAMP2* mutations (Figure 2F).

### EGCg Induces a Stronger Myofilament Ca^2+^ Desensitization in Strips From Patients With Truncating and Single Heterozygous Mutations

**Figure 3:**
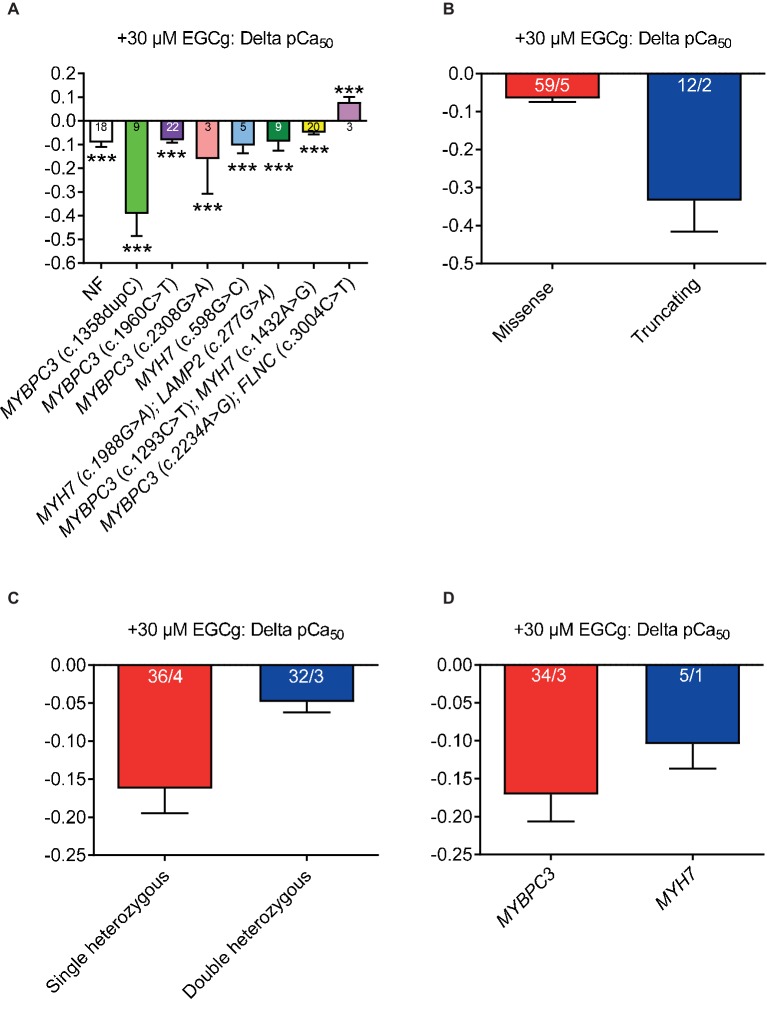
Effect of 30 μM EGCg on pCa_50_ values in permeabilized cardiac muscle strips isolated from seven HCM patients with either single heterozygous (*MYBPC3*, *MYH7)* or double heterozygous mutations (*MYH7/LAMP2*, *MYBPC3/MYH7*, *MYBPC3/FLNC)*. **(A)** Delta of pCa_50_ ± EGCg (30 μM) in non-failing control vs. HCM permeabilized cardiac muscle strips grouped according to mutation type (missense or truncating, **(B)**, to genetic status (single or double heterozygous mutations, **(C)**, and to a pure *MYBPC3*, *MYH7*, or *ACTN2* genotype **(D)**. Values are related to baseline. Data are expressed as mean ± SEM. ***p < 0.001 vs. baseline (without EGCg), paired Student’s t-test; for the different subgroup analyses, only qualitative comparisons were performed. *n* = number of strips; *n*/*N* = number of strips/number of patients as depicted in the bars.

We previously reported that EGCg decreased Ca^2+^ sensitivity of mouse and human cardiac myofilaments with *MYBPC3* mutations (Friedrich et al., 2016; Stucker et al., 2017). To assess whether the grade of desensitization is similar between HCM strips, we measured force-Ca^2+^ relationships in skinned ventricular muscle strips at baseline and after the application of 30 μM EGCg. Incubation (5 min) with EGCg shifted the force-Ca^2+^ curves to different extent to the right (pCa_50_ HCM_total_ + 30 μM EGCg: 5.54) of all strips (except in strips with double heterozygous *MYBPC3/FLNC* mutations) suggesting myofilament Ca^2+^ desensitization (Figure 3A). EGCg application tended to induce a more pronounced shift in strips from patients with truncating than missense (Figure 3B) and in strips with single than heterozygous mutations (Figure 3C). No major difference was observed between strips with *MYBPC3* or *MYH7* genotypes (Figure 3D). EGCg induced a force-Ca^2+^ curve right shift also in NF strips.

### HCM Samples Show a Heart Failure Gene Expression Profile

**Figure 4:**
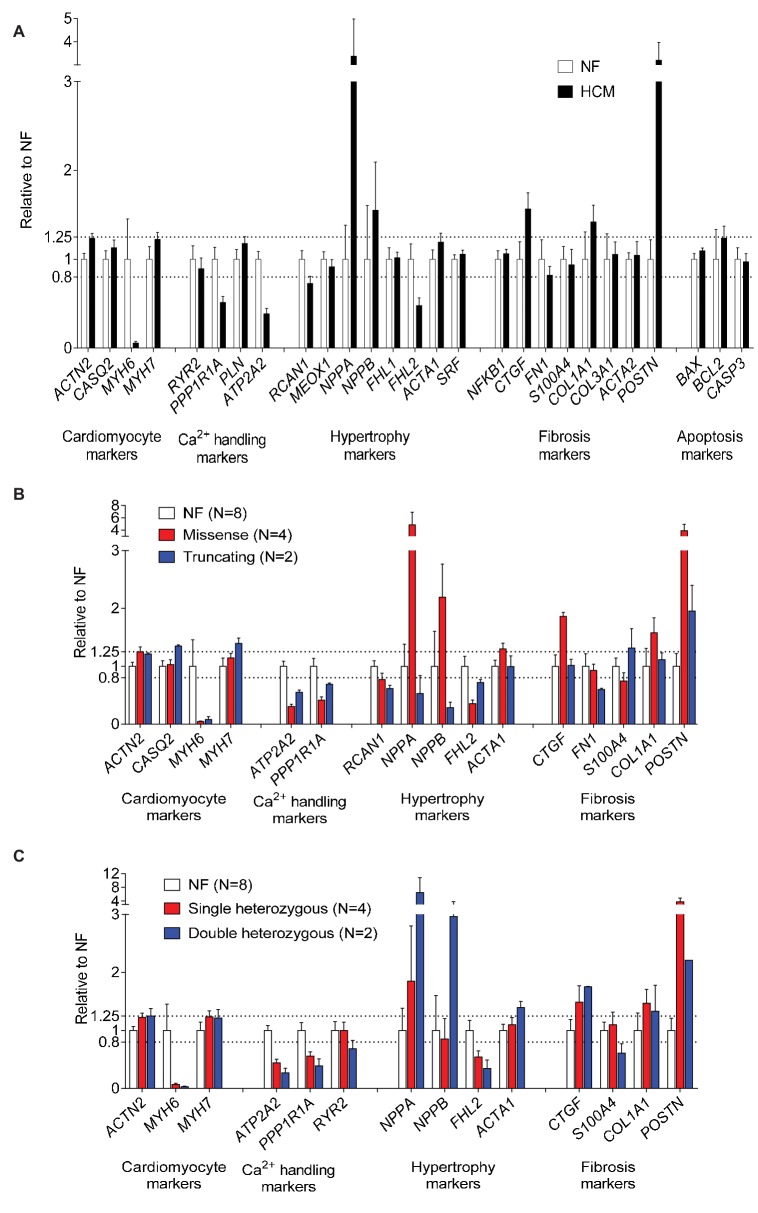
Evaluation of expression of human cardiac genes regulated in heart failure in NF and HCM tissues. Normalized expression levels of selected genes determined with the NanoString nCounter Elements technology comparing gene expression modulating hypertrophy, fibrosis, calcium handling, and apoptosis in NF and HCM **(A)**, (threshold <0.8- or > 1.25-fold change to NF). Control group consisted of RNA isolated from eight different NF control hearts. Gene expression related to **(B)** mutation type (missense or truncating) or **(C)** genetic status (single or double heterozygous mutation). Since for patient #7 (Table 1) obtained results repeatedly failed, quality control check data were not included in the analysis.

We then evaluated the expression levels of a customized panel of human genes (*n* = 27) regulated in heart failure using the NanoString nCounter® technology platform. Compared to NF samples (*n* = 8), gene expression analysis revealed lower *MYH6*, but higher *MYH7* and *ACTN2* mRNA levels, lower mRNA levels of Ca^2+^ handling proteins (*ATP2A2*, *PPP1R1A*), lower hypertrophy-associated *FHL2*, and higher *NPPA* and *NPPB mRNA* levels in HCM samples. Furthermore, mRNA levels of fibrosis markers (*COL1A1, CTGF, POSTN*) were higher (Figure 4A). Gene expression of markers related to hypertrophy (*NPPA*, *NPPB*, *ACTA1*) and fibrosis (*CTGF*, *COL1A1*, *POSTN*) were higher in tissues with missense than with truncating mutations (Figure 4B). mRNA levels of hypertrophy-associated *FHL2* were lower and of *NPPA* and *NPPB* were higher in tissues with double than with single heterozygous mutations (Figure 4C).

## Discussion

The observation of an increased myofilament Ca^2+^ sensitivity has mainly been made in HCM transgenic mouse models, reconstituted myofilament systems, and isolated cardiomyocytes, but data from multicellular tissues from HCM patients with mutations in different HCM genes have not been reported before (Morimoto et al., 1998; Tardiff et al., 1999; Cazorla et al., 2006; Pohlmann et al., 2007; Robinson et al., 2007; Jacques et al., 2008; Morimoto, 2008; van Dijk et al., 2009, 2012; Vignier et al., 2009; Huke and Knollmann, 2010; Kimura, 2010; Fraysse et al., 2012; Moore et al., 2012; Barefield et al., 2014; Flenner et al., 2016; Wijnker et al., 2016; Stucker et al., 2017; Ren et al., 2018). We therefore investigated force development and myofilament Ca^2+^ sensitivity in multicellular cardiac muscle strips derived from septal myectomies of patients with different HCM gene mutations and evaluated the potential use of epigallocatechin-3-gallate (EGCg), a known Ca^2+^-desensitizer, for myofilament Ca^2+^ desensitization.

The main findings of this study were as follows: (1) F_max_ tended to be higher in permeabilized cardiac muscle strips from patients with single heterozygous than with double heterozygous mutations and NF control, and in *MYBPC3* vs. *MYH7* strips. (2) Myofilament Ca^2+^ sensitivity was higher in HCM strips than in NF. (3) Myofilament Ca^2+^ sensitivity was greater by trend in samples with double than with single heterozygous mutations. (4) EGCg induced myofilament Ca^2+^ desensitization in almost all strips. EGCg effect was stronger by tendency in strips with truncating or single heterozygous mutations. (5) RNA expression analysis of a cardiomyopathy gene panel showed lower *MYH6*, but higher *MYH7* and *ACTN2* levels, lower levels of Ca^2+^ handling genes (*ATP2A2*, *PPP1R1A*), lower hypertrophy-associated *FHL2*, and higher *NPPA* and *NPPB* levels in comparison to NF samples. Furthermore, gene expression level differed between the genetic status (single or double heterozygous mutations) and between the mutation type (missense or truncating).

Force development had a tendency to be higher in cardiac muscle strips from patients with single heterozygous mutations or *MYBPC3* mutations, but did not differ to NF in samples carrying double heterozygous or *MYH7* mutations. Variable degrees of force development have been reported before. Most of the published data derived from *in vitro* studies with HCM-associated *MYH7* mutations are consistent with increased contractility (Debold et al., 2007; Palmer et al., 2008; Marston, 2011; Moore et al., 2012; Sommese et al., 2013; Spudich et al., 2016). However, a reduced F_max_ was also reported despite increased myofilament Ca^2+^ sensitivity (van Dijk et al., 2009). Force values for NF donor samples are lower than reported by other groups such as by Mamidi et al. (2017). One reason could be different types of measurement setups and another reason could be the difference in dimensions of the measured samples. Thinner preparations are more multicellular, whereas our samples contain also extracellular matrix which does not contribute to force generation.

As mentioned before, to this extent, data from multicellular tissues from HCM patients with mutations in different HCM genes have not been reported before. In this study, HCM patients’ strips derived from myectomies revealed a higher myofilament Ca^2+^ sensitivity than the NF control or showed at least a trend. An increased myofilament Ca^2+^ sensitivity leading to Ca^2+^ trapping and altered Ca^2+^ fluxes during excitation-contraction coupling is believed to play a central role in disease presentation (Ashrafian et al., 2011). Increased force development and Ca^2+^ sensitization can trigger hypertrophy, which leads to HCM phenotypic expression. Hypertrophy in turn can influence Ca^2+^ transients and other cellular processes such as energy metabolism, myofilament contraction, and electrophysiology. These processes boost hypertrophy and promote HCM disease expression. Clinically, all HCM patients showed an increased LV wall thickness. The increased myofilament Ca^2+^ sensitivity may also contribute to relaxation deficits and arrhythmias (Morimoto et al., 1998; Baudenbacher et al., 2008). An increased myofilament Ca^2+^ sensitivity is compatible with the compromised diastolic function and incomplete relaxation observed in HCM patients (Authors/Task Force et al., 2014) and in mice (Tardiff et al., 1999; Cazorla et al., 2006; Pohlmann et al., 2007; Vignier et al., 2009; Fraysse et al., 2012; Barefield et al., 2014). Five of the eight patients of this study presented a marked diastolic dysfunction. As for arrhythmias, four of the patients had episodes of ventricular tachycardia, which made an ICD implantation necessary.

Furthermore, myofilament Ca^2+^ sensitivity tended to be higher in strips with double than single heterozygous mutations and in strips with *MYBPC3* compared to *MYH7* mutations. An even higher Ca^2+^ sensitivity could exaggerate the consequences described above (Ca^2+^ trapping, increased force development…). Since the number of patients was low, interpretation of these data has to be treated with caution. The difference in Ca^2+^ sensitivity between single and double heterozygous mutations fits to the often-observed clinical difference in phenotypes. Patients with double mutations generally show an earlier disease presentation, more severe LV hypertrophy, higher prevalence of advanced heart failure, and an increased risk of sudden cardiac death than patients with single heterozygous mutations (Maron et al., 2012; Biagini et al., 2014; Fazeli Dehkordy et al., 2018). A differential Ca^2+^ sensitivity has also been observed in other studies with different HCM disease genes (Ren et al., 2018; Mamidi et al., 2019). It therefore seems that the direction and magnitude of the change not only depends on the affected gene (*MYBPC3* vs. *MYH7*) but also on the genetic status (single vs. double heterozygous), but not on the mutation type (missense vs. truncating).

Clinical management of HCM is challenging. Guidelines recommend treatment with beta blockers and Ca^2+^ channel antagonists, which improve clinical symptoms, moderately prevent arrhythmias, improve diastolic dysfunction, and reduce outflow tract obstruction (Tardiff et al., 2015). The idea of myofilament desensitization to reduce diastolic dysfunction and arrhythmias in HCM patients has not been tested clinically because of a lack of clinically applicable Ca^2+^ desensitizers. We and others reported that EGCg lowered myofilament Ca^2+^ sensitivity in mouse and human HCM tissues (Warren et al., 2015; Messer et al., 2016; Friedrich et al., 2016; Stucker et al., 2017). Additionally, Sheehan et al. and others reported that EGCg and structurally related compounds restored the coupled relationship between Ca^2+^-sensitivity and TnI phosphorylation in mutant thin filaments, which was independent of the underlying mutation (Papadaki et al., 2015; Messer et al., 2016; Sheehan et al., 2018). EGCg has been suggested to alter the interaction between cardiac troponin C and troponin I and thereby the sensitivity of the myofilaments to Ca^2+^ (Robertson et al., 2009). In the current study, we extended the analysis to several HCM muscle strips with differing HCM gene mutations and confirmed that application of EGCg shifted the force-Ca^2+^ curves of nearly all strips to the right, suggesting myofilament Ca^2+^ desensitization. EGCg application tended to induce a more prominent shift in strips with truncating than missense mutations or with single than double heterozygous mutations. Similar to the differential effects of EGCg on human HCM strip Ca^2+^ sensitivity in this study, we previously observed that EGCg had a more prominent effect on cardiac strips of an HCM *Mybpc3* knock-in (KI) mouse model than the WT control group (Friedrich et al., 2016). It is also compatible with results of a study in which the Ca^2+^ desensitizing effect of ranolazine was only present in KI, but not in WT muscle strips (Flenner et al., 2016). In contrast, EGCg induced a force-Ca^2+^ curve right shift also in NF strips. Thus, these differential EGCg effects could depend on the mutation, genetic status (single vs. double heterozygous), and the mutation type (missense vs. truncating).

Similar to the observations made in cardiomyopathy tissue with a reduced ejection fraction, we observed altered mRNA steady-state concentrations in myosin heavy chain isoforms (*MYH6/7*), heart-failure-associated Ca^2+^-handling (*ATP2A2*, *PPP1R1A*), hypertrophy (*FHL2*, *NPPA*, *NPPB*), and fibrosis (*COL1A1, CTGF, POSTN*) markers in comparison to NF samples. This is partially in accordance with previous findings using HCM samples, HCM human-induced pluripotent stem cells (iPSC)-CMs, or human embryonic stem cells carrying *MYBPC3* mutations as well as microarray results obtained in cardiac tissues of HCM patients prior to transplantation (Tanaka et al., 2014; Messer et al., 2016; Singh et al., 2017; Braumann et al., 2018; Mohammad et al., 2018). Furthermore, a marked difference in gene expression was detected between mutation type (missense vs. truncating) and genetic status (single vs. double heterozygous mutations). This study does not provide any explanation for this difference. As far as we are aware, such a discrepancy has not been reported before.

### Limitations of the Study

Although we were able to study a collection of human HCM samples with different mutations in genes encoding sarcomeric proteins, care must be taken when extrapolating our findings to all patients with HCM. Our HCM population consisted of patients with left ventricular outflow tract obstruction. As all HCM patients received a certain medication regime, we cannot exclude effects of medication on the outcome of our study. Due to the low numbers (each genotype *N*_patient_ = 1, subgroup analysis *N*_patient_ = 1–5), the data have to be treated with caution. We used a mixed-effect model analysis when analyzing all groups. To avoid type-II errors, we performed qualitative comparisons for the different subgroup analyses and discussed possible trends. Furthermore, sarcomere length could not be determined reliably in skinned HCM strips due to myocardial and myofilament disarray. Differences in sarcomere length could therefore have an effect on pCa_50_ values.

## Conclusion

We reported an increased myofilament Ca^2+^-sensitivity in native multicellular cardiac strips of HCM patients. Our findings suggest that myofilament Ca^2+^ desensitizing approaches might be useful for the treatment of HCM-associated diastolic dysfunction. Our data highlight that mutation-induced changes in myofilament Ca^2+^ sensitivity and response to EGCg are diverse and depend on the mutation, genetic status, and mutation type.

## Author Contributions

NK and SS contributed to isolation and treatment of cardiac muscle strips and execution of experiments. EK contributed to RNA isolation and execution of RNA expression experiments. GM and FF contributed to preservation of human cardiac tissues and database maintenance. JM and MP contributed to patients’ recruitment. CR contributed to genotyping of samples. LC is responsible for interpretation of data and correction of the manuscript. FWF contributed to conception and design of research, execution of experiments, analysis and interpretation of data, figure preparation, and drafting of the manuscript. All authors critically reviewed and approved the manuscript before submission.

### Conflict of Interest Statement

The authors declare that the research was conducted in the absence of any commercial or financial relationships that could be construed as a potential conflict of interest.

## Footnotes

^1^http://www.mutationtaster.org

^2^http://genetics.bwh.harvard.edu/pph2/
